# Unveiling the Effect of Ti Micro-Alloying on the Microstructure and Corrosion Resistance of the GH3536 Alloy Processed by Laser Metal Deposition in a Simulated Environment for PEMFCs

**DOI:** 10.3390/ma17235900

**Published:** 2024-12-02

**Authors:** Bing Xu, Bo Li, Jie Zhang, Jianping Tong, Yi Liu

**Affiliations:** 1 Key Laboratory of Quantum Precision Measurement of Zhejiang Province, School of Physics, Zhejiang University of Technology, Hangzhou 310023, China; b535763941@163.com (B.X.); libo@zjut.edu.cn (B.L.); liuyiphy@zjut.edu.cn (Y.L.); 2 College of Materials Science and Engineering, Taiyuan University of Science and Technology, Taiyuan 030024, China; zhangjie-zjut@foxmail.com

**Keywords:** corrosion behavior, GH3536 alloy, passive films, laser metal deposition, Ti addition

## Abstract

This article addresses the knowledge gap regarding the effect of Ti addition on the microstructure and corrosion behavior of the LMD-processed GH3536 alloy in a simulated solution of proton exchange membrane fuel cells (PEMFCs). The microstructural evolution, corrosion resistance, and passive film characteristics of LMD-processed GH3536 alloy with varying Ti contents were characterized through a variety of techniques, including scanning electron microscopy (SEM), X-ray diffraction (XRD), transmission electron microscopy (TEM), energy dispersive spectroscopy (EDS), electron backscatter diffraction (EBSD), X-ray photoelectron spectroscopy (XPS), and a series of electrochemical measurements. The results indicate that the corrosion resistance of the LMD-processed GH3536 alloy significantly improves with increasing Ti content. However, when the Ti content exceeds 0.2 wt.%, the beneficial effect on corrosion resistance is weakened. Two primary mechanisms explain the enhanced corrosion resistance, involving the heterogeneous nucleation of Ti-modified Al_2_O_3_ and Ti solute segregation, which promotes grain refinement. In addition, grain refinement can provide more active sites for the formation of compact passive films, thereby improving corrosion resistance of the GH3536 alloy.

## 1. Introduction

There are two major challenges for humanity, involving energy crisis and environmental pollution, with an urgent need to explore new green energy sources [1]. Proton-exchange membrane fuel cells (PEMFCs) are considered a pivotal technology in renewable energy systems, owing to their excellent energy conversion efficiency and environmentally friendly emissions [2]. These fuel cells have been broadly adopted across various fields, such as new energy vehicles, aerospace, and a range of other industries [3,4]. A key factor that significantly improves the performance and efficiency of PEMFCs is bipolar plates (BPs). This component constitutes at least 80% of the stack’s weight and volume and is responsible for about 40% of the total production cost related to PEMFCs [5,6].

As a fundamental structural component of fuel cells, the bipolar plate features a well-engineered flow field that significantly optimizes the mass transfer and distribution of gas and liquid within the PEMFC’s flow channels. In addition, this innovative design also enhances heat dissipation, effectively reducing the risk of liquid flushing [1,6]. To successfully commercialize fuel cells and make them competitive with alternative energy sources, it is essential to improve their durability and lower production costs. However, bipolar plates made from composite materials and graphite face considerable limitations due to their poor mechanical properties and high manufacturing costs [7].

In this context, GH3536, a prominent nickel-based alloy, emerges as a promising candidate for bipolar plate applications. This alloy is primarily strengthened by chromium (Cr) and molybdenum (Mo), and possesses an outstanding corrosion resistance, superior mechanical properties, and structural stability across a wide temperature range [8,9]. It is crucial to acknowledge that conventional manufacturing methods encounter significant obstacles in meeting the complex flow channel designs and precision requirements of bipolar plate components [10]. In recent years, additive manufacturing (AM) technology has progressed remarkably [11], with laser metal deposition (LMD) emerging as a valuable technique that facilitates the direct fabrication of highly dense, intricately structured, and high-performance metal components. However, it is important to consider the increase in the corrosion susceptibility of alloys produced by this technique, especially when compared to those prepared by traditional forging methods [12]. Another AM technique which has been evaluated as an option to reduce BP manufacturing costs is cold spray additive manufacturing (CSAM), which is a high deposition rate solid state technique that prevents degradation of the feedstock powder due to its low-temperature processing [13]. For example, the extreme conditions associated with a rapid melting and solidification, as well as a cyclical heating and cooling in the deposition process causes a unique microstructure, such as the segregation of M_23_C_6_ and molybdenum-rich phases along the grain boundaries (GBs) [14,15].

In the PEMFCs, the dissolved metal ions in weakly acidic media along with cathodic reactant gasses can contaminate the membrane electrode assembly, leading to a decrease in the efficiency of PEMFCs [16]. Consequently, researchers have primarily focused on optimizing process parameters [17,18] and applying post-heat treatments [19,20] to improve the corrosion resistance and other properties of alloys produced through the LMD method. It is essential to note that the efficacy of these methods is limited by the continuous re-melting and cooling, as well as potentially localized high temperatures during the operation. As a result, many researchers have dedicated their efforts towards modifying alloy compositions to improve their performance, particularly with respect to corrosion resistance. For instance, Zhang et al. investigated the effects of niobium (Nb) alloying on the passive film and corrosion characteristics of the cobalt–chromium–iron–manganese–nickel (CoCrFeMnNi) high entropy alloy (HEA) produced through laser powder bed fusion (LPBF). Their research revealed that the addition of Nb can significantly improve the corrosion resistance of the HEA by optimizing the grain size, the type of precipitation phases and inclusions, as well as the composition of passive films [21]. Similarly, the addition of niobium (Nb) into titanium–aluminum (TiAl) alloys has been demonstrated to significantly improve their oxidation resistance, which can be attributed to the increase in twin boundaries and the formation of a continuous AlNb_2_ barrier layer [22]. Additionally, research by Chyrkin et al. [23] reveals that Si alloying effectively eliminates the intergranular oxidation in nickel-based alloy 625 produced through AM, and it is primarily due to the formation of SiO_2_ layers. Furthermore, Liu et al. [24] identified a novel synergistic interaction arising from high concentrations of Cr and Al elements, demonstrating that hydration reactions of Cr and Al corrosion-resistant elements significantly reduce the dissolution of Fe and Mn. On the other hand, numerous studies have thoroughly explored the incorporation of various nanoparticles, such as Y_2_O_3_ [25,26], TiB_2_ [27,28], TiC [29], CeO_2_ [30], and carbon nanotubes, to enhance material’s properties. In addition, Garfias et al. compared the use of a less costly irregular Ti instead of a spherical one to make BP by CSAM [13]. However, there is limited information available on the corrosion resistance improvement in the LMD-processed GH3536 alloy through Ti alloying. It is essential to note that the strong affinity between Ti and C promotes the precipitation of TiC, which may diminish the bonding interaction between Cr and C atoms at grain boundaries [31]. Moreover, the addition of Ti has been shown to positively impact grain refinement. Based on the grain boundary engineering (GBE) principle, the grain refinement can introduce special boundaries (SBs), such as low coincidence site lattice (CSL) boundaries (Σ29) and twin boundaries (Σ3), which is favorable to mitigate intergranular failure in materials [32]. Understanding the mechanistic role of Ti alloying influencing the corrosion resistance of LMD-processed GH3536 alloy is crucial; however, there is a lack of sufficient studies in this area.

Building on the aforementioned considerations, this article aims to address the existing knowledge gap regarding the corrosion resistance of the GH3536 alloy processed by LMD with varying Ti concentrations in a simulated environment of PEMFCs. The microstructural evolution, corrosion resistance, and passive film characteristics of LMD-processed GH3536 alloy with varying Ti contents were monitored using a variety of techniques, including scanning electron microscopy (SEM), X-ray diffraction (XRD), transmission electron microscopy (TEM), energy dispersive spectroscopy (EDS), electron backscatter diffraction (EBSD), X-ray photoelectron spectroscopy (XPS), and electrochemical measurements.

## 2. Experimental

### 2.1. Materials and Samples Preparation

**Figure 1:**
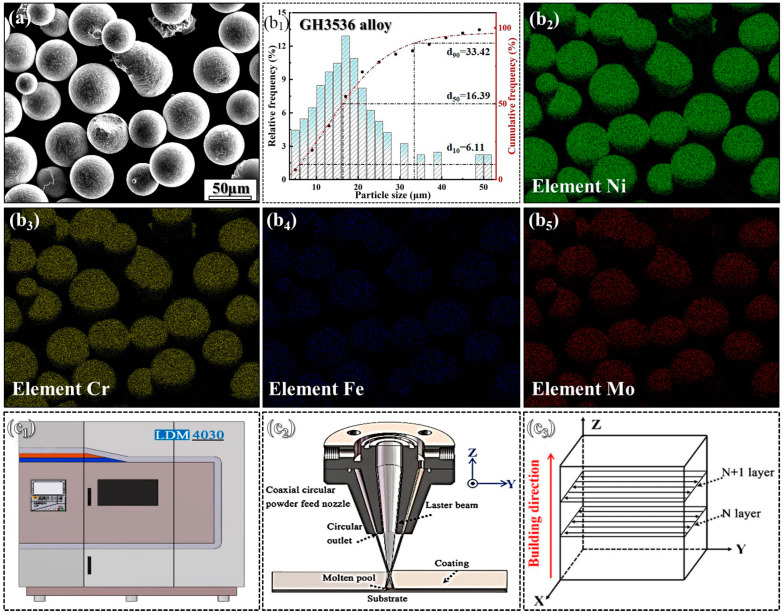
(**a**,**b_1_**–**b_5_**) SEM morphology, particle diameter, and element distribution of GH3536 alloy powders and (**c_1_**–**c_3_**) the corresponding schematic illustration of the LMD process [15,33].

Argon-atomized GH3536 alloy powders (≥99.99% purity) produced by Tianjin Zhujin Technology Development Co., Ltd. (Tianjin, China) were used as the base material for this investigation. The base metal to support the building LMD GH3536 deposit was 304 austenitic stainless steel. The detailed composition of the GH3536 powder is provided in Table 1, which is measured by using an Avio 200 inductively coupled plasma optic emission spectrometry (ICP-OES, PerkinElmer, Inc., Singapore) instrument at a forward power of 1.20 kW and plasma flow 15.0 L/min. Figure 1 illustrates the powder morphology with an excellent sphericity. The particle size distribution of powders is characterized by D_10_ = 6.11 μm, D_50_ = 16.39 μm, and D_90_ = 33.42 μm. Additionally, the Ti powders used in this study possess a purity level of 99.999%. Different amounts of Ti were introduced into the same system, with concentrations of 0.0, 0.1, 0.2, and 0.3 wt.%. To achieve a uniform mixture, the GH3536 alloy and Ti powders underwent mechanical blending using ball milling (with a ball-to-powder ratio of 5:1) for 60 min in an argon (Ar) atmosphere. The LMD process was carried out with an LMD machine (YLR-2000, IPG Photonics Corporation, Yangzhong, China) equipped with a powder feeder, cooling system, travel mechanism, and other essential components. Argon was utilized as the shielding gas, and the substrate was preheated to 100 °C. The resulting samples were fabricated as a straight-wall structure measuring 80 × 50 × 5 mm^3^.

### 2.2. Microstructual Characterization

XRD tests were performed to evaluate the crystalline structure of materials. To assess the influence of varying Ti concentrations on the microstructure, EBSD analysis was conducted using a SEM (Zeiss Merlin, Oberkochen, Germany) equipped with an Oxford NordlysMax3 detector. This analysis was performed with an acceleration voltage of 15 kV and a scanning step size of 1.6 µm. Prior to the tests, the samples underwent mechanical polishing and electrochemical polishing to ensure a smooth surface. EBSD data were subsequently processed and analyzed using an AztecCrystal 2.1 software.

### 2.3. Electrochemical Measurements

Electrochemical measurements were performed using a Gamry Reference 600 electrochemical workstation with a standard three-electrode cell setup. The test solution comprised 0.5 mol/L H_2_SO_4_ and 5 mg/L fluoride ions at a temperature of 25 °C, with air bubbling introduced to replicate the cathodic conditions in PEMFCs. The purpose of adding 5 mg/L fluoride ions (sodium fluoride salt) is to simulate the cathodic environment of PEMFCs. Before conducting the measurement, a constant potential of −1.2 V versus saturated calomel electrode (SCE) was applied for 5 min to remove the oxide layer that formed on the sample surface. The samples were then submerged in the electrolyte until a stable open-circuit potential (OCP) was achieved. The potential range of potentiodynamic polarization tests is from −0.8 V_SCE_ to 1.0 V_SCE_ at a scanning rate of 0.167 mV/s. Electrochemical impedance spectroscopy (EIS) measurements were conducted over a frequency range from 100 kHz to 10 mHz using an AC amplitude of 10 mV. To assess the stability of the passive films under PEMFC conditions, a potentiostatic polarization test was carried out at 0.5 V_SCE_ for 1 h. Mott–Schottky (M-S) measurements with a voltage range of −0.6 to 1.5 V_SCE_ were conducted after the potentiostatic polarization at a scanning rate of 50 mV/s. Additionally, double loop electrochemical potentiokinetic reactivation (DL-EPR) tests were performed to qualitatively evaluate the degree of sensitization (DOS) of samples, which was conducted from −0.5 to 0.5 V_SCE_ in a solution of 0.5 mol/L H_2_SO_4_ and 0.01 mol/L KSCN at 30 °C [34].

### 2.4. XPS Characterization

The chemical composition of passive films on the LMD-processed GH3536 alloy samples was assessed using an XPS with a Thermo Fisher ESCALAB 250Xi instrument. Prior to tests, a voltage of 0.5 V_SCE_ was applied to the sample surface for a period of 6 h.

## 3. Results

### 3.1. Microstructure

**Figure 2:**
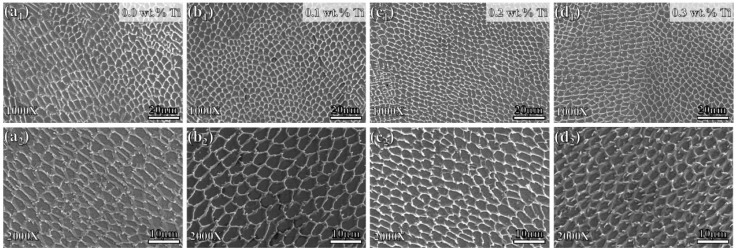
Microstructures of LMD-processed GH3536 alloy samples with various Ti contents: (**a_1_**,**a_2_**) 0.0 wt.%, (**b_1_**,**b_2_**) 0.1 wt.%, (**c_1_**,**c_2_**) 0.2 wt.%, and (**d_1_**,**d_2_**) 0.3 wt.%.

Figure 2 illustrates the microstructure of GH3536 alloy samples processed by LMD with varying Ti concentrations. The micrographs reveal well-refined cellular substructures along with distinct molten pools. During the LMD process, the laser beam generates a molten pool by melting the powder, which quickly solidifies at a high cooling rate of 10^6^ and 10^8^ K/s. The rapid movement of the laser beam leads to a steep local temperature gradient within the molten pool, causing the formation of overlapping elliptical pools. Notably, as the Ti content increases, the size of these cellular structures tends to decrease.

**Figure 3:**
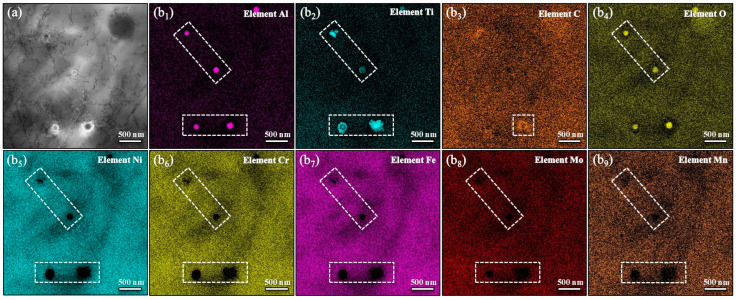
TEM micrographs (**a**) and the corresponding local EDS mapping images (**b_1_**–**b_9_**) of the Ti-modified inclusion.

**Figure 4:**
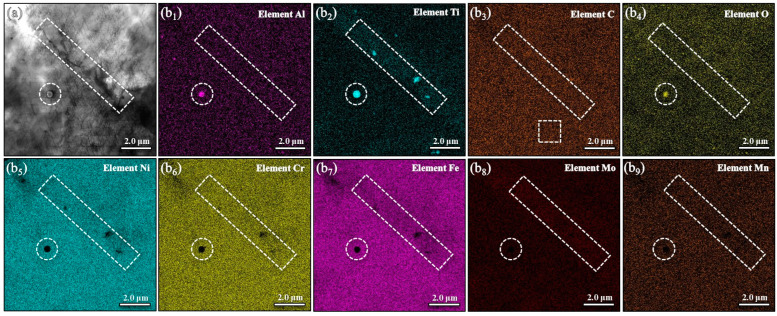
TEM micrographs (**a**) and the corresponding local EDS mapping images (**b_1_**–**b_9_**) of the segregation of Ti solute at the grain boundary.

To further explore the impact of Ti addition on the microstructure, TEM and corresponding local elemental analyses are provided in Figure 3 and Figure 4. Within the grain interior, nano-sized inclusions are observed, primarily consisting of Al and Ti elements. It is important to note that aluminum is primarily concentrated within the spherical inclusion, which is considerably smaller than the distribution of Ti elements. This phenomenon can be attributed to the formation of Al_2_O_3_ particles serving as heterogeneous nucleation sites for Ti compounds, as shown in Figure 3, resulting in the creation of Ti-modified Al_2_O_3_ particles that are uniformly dispersed within the deposition pool. Moreover, Figure 4 describes the segregation of the Ti solute at GBs, which plays a significant role in restraining grain growth and establishing a zone of constitutional supercooling.

**Figure 5:**
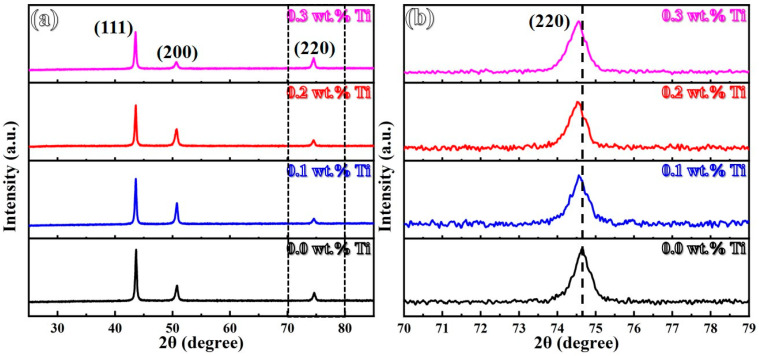
X-ray diffraction data of LMD-processed GH3536 alloy samples with various Ti contents: (**a**) 2θ = 25–85° and (**b**) 2θ = 70–79°.

Figure 5 displays XRD results of LMD-processed GH3536 alloy samples with varying Ti contents. As shown in Figure 5a, all samples exhibit a single face-centered cubic (FCC) structure, with distinctive reflection peaks at (111), (200), and (220) within the 25–85° angular range. This indicates that the introduction of Ti does not change the fundamental FCC structure of the LMD-processed GH3536 alloy. Notably, when comparing the XRD patterns of the original sample, a slight shift in the (220) peak towards a lower angle is recorded with increasing Ti contents. This shift is primarily ascribed to an increase in lattice parameters, particularly visible at 0.2 and 0.3 wt.% Ti [35].

**Figure 6:**
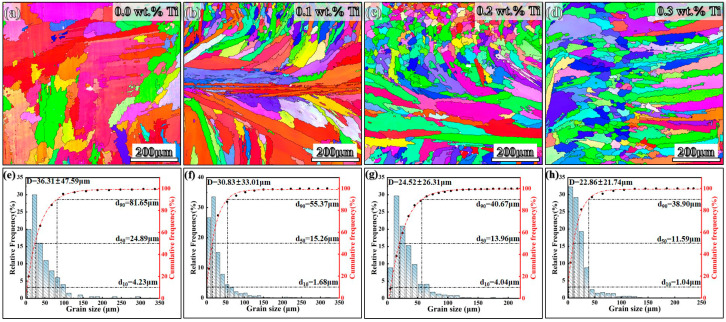
EBSD inverse pole figures (**a**–**d**) and the corresponding grain sizes (**e**–**h**) of LMD-processed GH3536 alloy samples with various contents of Ti: (**a**,**e**) 0.0 wt.%, (**b**,**f**) 0.1 wt.%, (**c**,**g**) 0.2 wt.% and (**d**,**h**) 0.3 wt.% [33].

**Figure 7:**
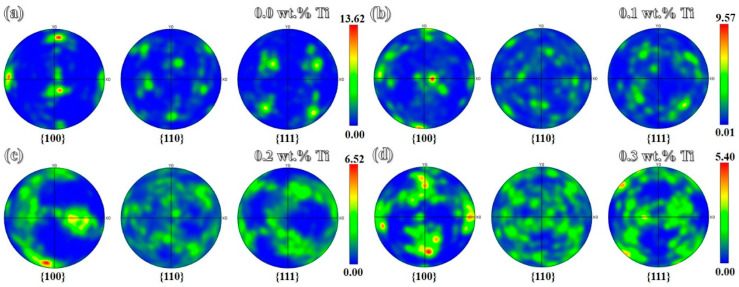
EBSD pole figures of LMD-processed GH3536 alloy samples with various contents of Ti: (**a**) 0.0 wt.%, (**b**) 0.1 wt.%, (**c**) 0.2 wt.% and (**d**) 0.3 wt.%.

**Figure 8:**
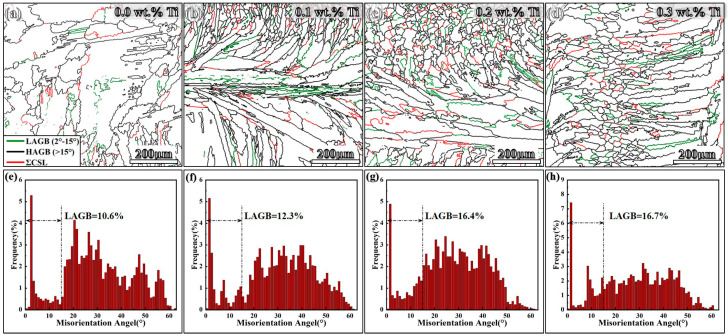
EBSD image quality with grain boundary plots (**a**–**d**) and the corresponding LAGB frequency (**e**–**h**) of LMD-processed GH3536 alloy samples with various contents of Ti: (**a**,**e**) 0.0 wt.%, (**b**,**f**) 0.1 wt.%, (**c**,**g**) 0.2 wt.%, and (**d**,**h**) 0.3 wt.%.

Figure 6 and Figure 7 present the inverse pole figure (IPF) and pole figure (PF) maps obtained from EBSD analysis. The IPF images depicted in Figure 6a–d reveal that the original sample displays coarse columnar grains. This structure arises from a high thermal gradient (G) to solidification rate (R) ratio during the LMD process, resulting in an average grain size of 36.31 µm^2^. In contrast, the grain size significantly decreases with increasing Ti contents, leading to the formation of equiaxed grains. The statistical grain size distribution analysis in Figure 6e–h indicates that the average grain size of LMD-processed GH3536 alloy samples with 0.1, 0.2, and 0.3 wt.% Ti decreases to 30.83 µm, 25.42 µm, and 22.86 µm, respectively. This decrease signifies that the incorporation of Ti into GH3536 alloy samples has a substantial effect on transforming the microstructure from predominantly columnar grains to equiaxed grains. Moreover, the PF results illustrated in Figure 7 indicate that the samples exhibit a distinct texture. The {100} pole figure of the original sample reveals a <001> solidification fiber texture, characterized by a peak texture index of 13.62. This high texture index is attributed to an epitaxial growth of columnar grains, which is commonly associated with the directional solidification in FCC alloys. However, this preferential orientation decreases significantly with the addition of Ti. Specifically, the maximum texture index of samples containing 0.1, 0.2 and 0.3 wt.% Ti are reduced to 9.57, 6.52, and 5.40, respectively. Figure 8 presents the GB characteristics of four samples. Low angle grain boundaries (LAGBs) are represented by green lines, while high angle grain boundaries (HABGs) are indicated by black lines. Additionally, low CSL boundaries, outlined in red, are defined according to Brandon [36], categorizing low CSL grain boundaries with Σ ≤ 29 as SGBs [37,38].

As shown in Figure 8a–d, the original LMD-processed GH3536 alloy sample has a minimal ratio of LAGBs and ΣCSL grain boundaries; however, as the Ti content increases, the proportion of these grain boundaries correspondingly rises. Previous studies have indicated that the variations in crystal structure can significantly impact the corrosion resistance of these grain boundaries [39]. The presence of low ΣCSL grain boundaries, characterized by a high density of junction points, can hinder the network of LAGBs. Their periodic atomic arrangement contributes to the decrease in the grain boundary energy, and, thus, enhances the material’s corrosion resistance [40,41]. Additionally, it is noteworthy that LAGBs and ΣCSL boundaries have significantly lower energy compared to HABGs, further improving the corrosion resistance of alloys.

### 3.2. Potentiodynamic Polarization

**Figure 9:**
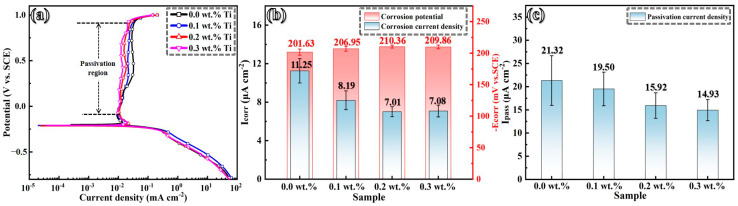
Potentiodynamic polarization curves (**a**) and obtained electrochemical parameters (**b**,**c**) of LMD-processed GH3536 alloy samples with various contents of Ti in a simulated PEMFC solution at 25 °C.

Figure 9a presents the potentiodynamic polarization curves for the GH3536 alloy fabricated by LMD in a simulated PEMFC solution. These curves clearly illustrate that, with increasing the applied voltage, the samples display a typical passive behavior. This indicates that the addition of Ti does not fundamentally change the electrochemical characteristic of the GH3536 alloy. Figure 9b,c depicts the corrosion potential (*E*_corr_), corrosion current density (*i*_corr_), and passivation current density (*i*_pass_). Compared to the original sample, the *E*_corr_ value exhibits a decline with increasing Ti concentrations. This decrease is primarily attributed to the formation of additional galvanic cells after the Ti addition, which induces a preferential anodic dissolution during the initial corrosion stage. The *i*_corr_ value tends to decrease with a higher Ti content, implying the increase in the corrosion resistance of GH3536 alloy samples. However, it is crucial to recognize that *i*_corr_ alone should not be used exclusively to evaluate corrosion resistance due to the interaction between passivation and activation [42]. According to previous research [43,44], *i_pass_* serves as an indicator of the dissolution rate of passive films during the passive stage. A higher passive current density at a higher Ti content correlates with an increased dissolution rate of passive films under a simulated PEMFC condition. This indicates that the corrosion resistance of passive films formed on the LMD-processed GH3536 alloy samples improves progressively with increasing Ti contents.

### 3.3. Electrochemical Impedance Spectroscopy

**Figure 10:**
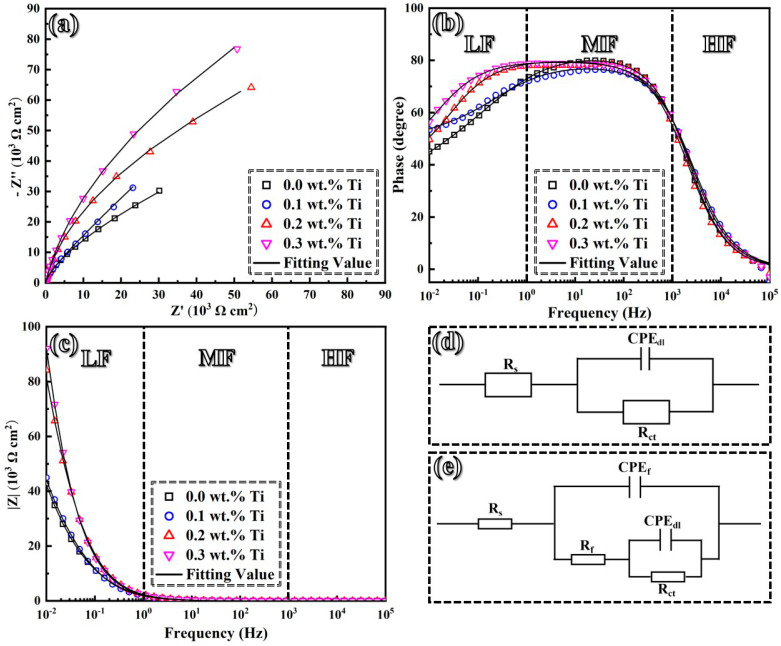
EIS curves (**a**–**c**) and the corresponding equivalent circuits (**d**,**e**) of LMD-processed GH3536 alloy samples with various contents of Ti in a simulated PEMFC solution at 25 °C.

As previously documented, the LMD-processed GH3536 alloy can form a passive film when it is immersed in a diluted sulfuric acid solution [45]. To investigate the effect of Ti addition on the stability of passive films, EIS tests were conducted, as shown in Figure 10. All samples display similar capacitive loops, characterized by an incomplete semicircle, as illustrated in Figure 10a. Notably, the increase in Ti contents leads to a larger diameter of capacitive semicircles, indicating enhanced corrosion resistance of passive films. Additionally, Figure 10b reveals a linear relationship between log |Z| and log f in the low-frequency region, which indicates that the capacitive response dominates at the electrode/solution interface [46]. In Figure 10c, the log |Z| value in the low-frequency range significantly increases with the Ti content from 0.0 wt.% to 0.2 wt.%, while this trend becomes inapparent when the Ti content reaches 0.3 wt.%. The phase angle curve suggests that the time constants related to passive films and the charge transfer process overlap, resulting in a horizontal plateau in the mid- to low-frequency ranges. No significant changes in the phase angle are noted when the Ti content ranges from 0.0 to 0.1 wt.%. However, this horizontal plateau broadens and rises, indicating a more stable structure of passive films [47].

The EIS data were accurately fitted using the equivalent circuit R(QR) for samples containing 0.0 and 0.1 wt.% Ti, while the equivalent circuit R(Q(R(QR))) was used for samples with 0.2 and 0.3 wt.% Ti, as illustrated in Figure 10d,e. The fitting results are summarized in Table 2. In this analysis, *R*_s_, *R*_f_, and *R*_ct_ represent the electrolyte resistance, film resistance, and charge transfer resistance, respectively. *CPE*_f_ and *CPE*_dl_ denote the capacitances of passive films and the double layer, while n_f_ and n_dl_ correspond to the exponents of constant phase elements (*CPE*) associated with the double layer [48,49]. The impedance of the *CPE* is defined by Equation (1).

(1) $$Z_{CPE}=\frac{1}{Y_{0}}(j\omega {)}^{-n}$$

In this equation, *Y*_0_ signifies a magnitude of the CPE, while ω = 2π*f* represents an angular frequency (with f being a standard frequency in Hz), and j is an imaginary unit. The dispersion coefficient, n, reflects surface heterogeneity [50]. The polarization resistance (*R*_p_), defined as a sum of *R*_f_ and *R*_ct_, is commonly utilized to assess the corrosion resistance of samples in corrosive environments, showing an upward trend with increased Ti contents.

### 3.4. Potentiostatic Measurements

**Figure 11:**
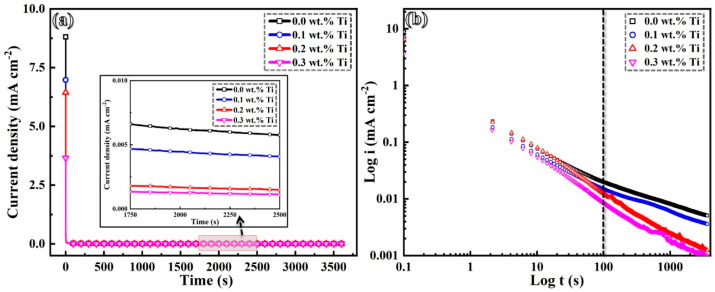
Steady-state passive current density (**a**) of LMD-processed GH3536 alloy samples with various contents of Ti under a potential of 0.5 V vs. SCE in a simulated PEMFC solution at 25 °C and corresponding double-logarithmic plots of current–time (**b**).

Figure 11a illustrates the potentiostatic polarization curves of GH3536 alloy samples that were processed by LMD in a simulated PEMFC solution at 25 °C, with a potential set at 0.5 V_SCE_. Initially, there is a notable decrease in current density, which can be attributed to the formation and subsequent thickening of passive films [51]. The current densities for the samples with 0.2 and 0.3 wt.% Ti stabilize at lower levels, indicating the presence of more compact passive films. The relationship between the current density and time is further defined by a double-logarithmic Equation (2) [51]:

(2) $$i=10^{-(b+klogt)}$$

where *i* represents the current density, *t* stands for time, and k and b are constants. A slope of k = −1.0 indicates the formation of a highly protective surface film that is controlled by high field parameters, whereas a slope of *k* = −0.5 suggests the creation of a diffusion-controlled surface film with a lower protective efficiency [52,53]. From the double-logarithmic plots displayed in Figure 11b and summarized in Table 3, it is evident that the *k* value for the original sample begins at −0.61 in the early stage and increases to −0.37 in the later stages. The initial slope values for the samples containing 0.2 and 0.3 wt.% Ti are −0.79 and −0.78, respectively, both of which rise to −0.60 and −0.59 with time. These variations in slope values imply that the passive films of samples with 0.0 and 0.1 wt.% Ti are initially compact and become increasingly porous as time progresses. In contrast, the passive films formed on the samples containing 0.2 and 0.3 wt.% Ti maintain a consistently compact structure.

### 3.5. Mott–Schottky Analysis

**Figure 12:**
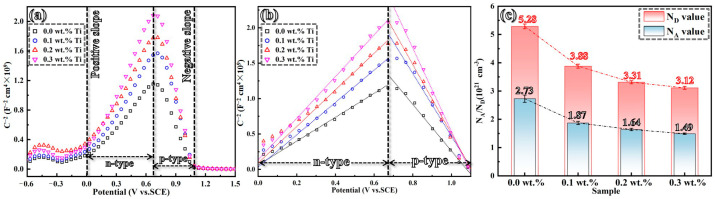
Mott–Schottky plots (1000 Hz) of LMD-processed GH3536 alloy samples with various contents of Ti in a simulated PEMFC solution at 25 °C (**a**,**b**) and the corresponding variations (**c**) in the defect concentration.

Figure 12 presents the Mott–Schottky curves of passive films formed on four GH3536 alloy samples processed by LMD in a simulated PEMFC solution at 25 °C. As anticipated, all Mott–Schottky plots exhibit two distinct regions, which are characterized by linear segments above and below a potential of approximately −0.68 V_SCE_. For nickel-based alloys, it is well-recognized that their semiconducting behavior reflects the dual composition of surface films, which consists of an inner layer primarily composed of Cr oxides and an outer layer predominantly made up of Fe/Ni oxides. The positive slope of curves (Region I) indicates that passive films behave as an n-type semiconductor, in which oxygen vacancies and cation interstitials serve as electron donors and are primary charge carriers. Conversely, Region II features a negative slope, suggesting that passive films function as a p-type semiconductor, where cation vacancies act as electron acceptors and are main charge carriers [54]. As highlighted in previous research [55], the slopes of the linear sections of Mott–Schottky plots can be described by the equations of 2/(εε_0_eN_D_) for n-type oxide films and −2/(εε_0_eN_A_) for p-type oxide films. The donor concentration (N_D_) and the acceptor concentration (N_A_) can, thus, be calculated. It is important to note that fluctuations in N_D_ and N_A_ concentrations do not directly correlate with impurity levels but rather suggest the presence of non-stoichiometric defects within the space charge region or highlight the disordered structure of passive films [56,57]. Both N_D_ and N_A_ follow a similar trend, reaching lower values of 3.31 × 10^21^ cm^−3^ (1.64 × 10^21^ cm^−3^) and 3.12 × 10^21^ cm^−3^ (1.49 × 10^21^ cm^−3^), respectively, at Ti contents of 0.2 wt.% and 0.3 wt.%. Given that the N_A_ values obtained from the slopes in Region II are lower, this study emphasizes the N_D_ values of passive films, as depicted in Figure 12b,c. According to the Point Defect Model (PDM) [58], the N_D_ reflects the attraction of corrosive ions to passive films. A decrease in N_D_ is strongly related to a decrease in point defects within passive films. Furthermore, a more compact passive film correlates with an enhanced corrosion resistance, which can be effectively achieved by the addition of Ti in the alloy.

### 3.6. DL-EPR Tests

**Figure 13:**
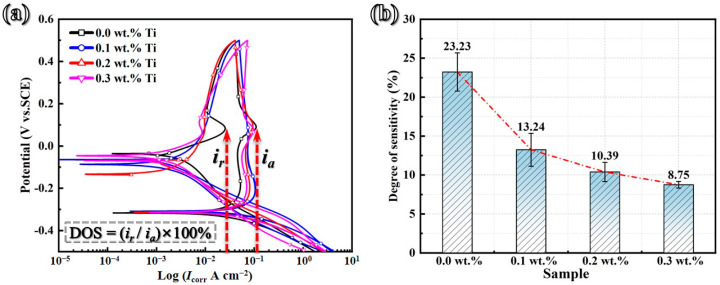
DL-EPR curves (**a**) of LMD-processed GH3536 alloy samples with various contents of Ti in a 0.5 M H_2_SO_4_ + 0.01 M KSCN solution at 25 °C and the corresponding variations (**b**) in the DOS values.

Previous research has shown that the GH3536 alloy processed by LMD is particularly susceptible to severe IGC in diluted sulfuric acid solutions [15]. To assess the impact of Ti addition on IGC resistance, DL-EPR tests of Ti-modified GH3536 alloy samples were conducted, as illustrated in Figure 13a. The findings reveal that during the early stage of anodic polarization, the current density exhibits an initial increase, reaching a peak value. Following this peak, the current density slightly decreases and stabilizes at a lower level, indicating an occurrence of passivation. Importantly, the peak activation current density (*i_a_*) remains largely unchanged with the addition of Ti. Upon reversing the potential scan, the curve displays instability, with a weaker current density peak that is recognized as the reactivation current density peak (*i_r_*). The observed potential difference between ia and ir can be attributed to an increase in the film’s resistance resulting from the initial polarization process [59,60]. The relationship between the DOS values (DOS = (*i_r_*/*i_a_*) × 100%) and the Ti content was calculated and illustrated in Figure 13b. The results show a significant decrease in DOS values from 23.23% to 10.39% with the addition of 0.2 wt.% Ti. Moreover, as the Ti content is further increased to 0.3 wt.%, the variation in DOS values remains relatively minor, fluctuating between 10.39% and 8.75%. These observations suggest that the IGC resistance of the Ti-modified GH3536 alloy processed by LMD can be effectively enhanced when the Ti content reaches 0.2 wt.%. As the Ti concentration rises from 0.2 wt.% to 0.3 wt.%, the positive impact of Ti on corrosion resistance appears to be weakened.

### 3.7. XPS Analysis

**Figure 14:**
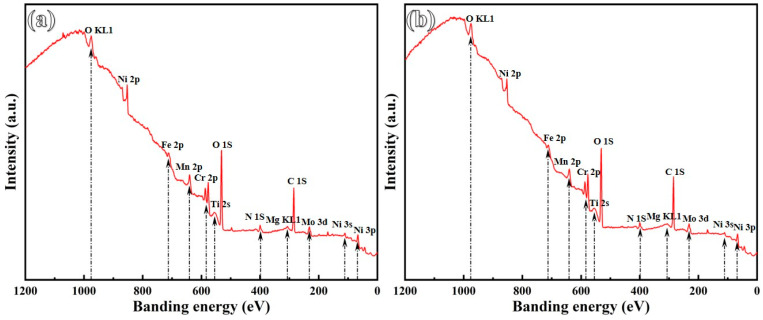
High-resolution XPS spectra (survey spectrum) of LMD-processed GH3536 alloy samples with (**a**) 0.0 wt.% and (**b**) 0.2 wt.% Ti after a 6 h of potentiostatic polarization tests in a simulated PEMFC solution at 25 °C.

**Figure 15:**
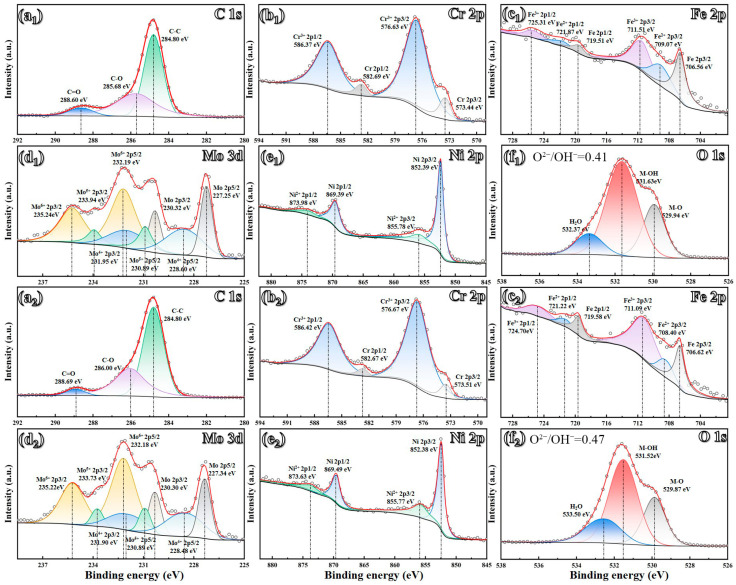
High-resolution XPS spectra (C 1s, O 1s, Cr 2p, Ni 2p, Fe 2p and Mo 3d) of LMD-processed GH3536 alloy samples with (**a_1_**–**f_1_**) 0.0 wt.% and (**a_2_**–**f_2_**) 0.2 wt.% Ti after a 6 h of potentiostatic polarization tests in a simulated PEMFC solution at 25 °C.

**Figure 16:**
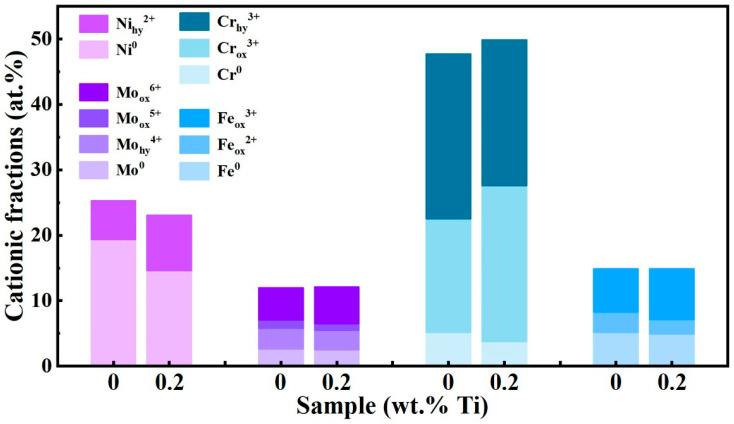
The cationic fractions in the passive films of LMD GH3536 alloy samples with 0.0 wt.% and 0.2 wt.% Ti, respectively.

The impact of Ti addition on the composition of passive films formed on the GH3536 alloy processed by LMD in a simulated PEMFC solution was investigated using an XPS. The results indicate that all passive films contain Fe, Cr, Ni, and Mo oxides. After subtracting the Shirley baseline, the high-resolution XPS spectra were analyzed, as shown in Table 4 and Figure 14 and Figure 15. The Fe 2p spectrum was resolved into several distinct peaks, which represent the metallic form (Fe_(met)_) as well as oxidation states of Fe^2+^ and Fe^3+^ [61]. Similarly, the Cr 2p spectrum displays the peaks corresponding to the metallic state (Cr_(met)_), Cr oxide (Cr_2_O_3_), and Cr hydroxide (Cr(OH)_3_) [62]. Previous studies [63] have indicated that Cr_2_O_3_, characterized by a lower concentration of point defects like cation vacancies, is more stable than Cr(OH)_3_. The formation of passive films is primarily linked to the selective dissolution of Fe during corrosion, which leads to Cr enrichment within passive films. As shown in Figure 16, the proportion of Cr_2_O_3_ in the passive film increases with a higher Ti content, largely due to grain refinement, which provides more active sites conducive to the formation of Cr_2_O_3_. The peak intensity of metallic Cr noticeably decreases following Ti addition, suggesting an easier reaction of Cr with oxygen compared to the original sample [64]. Analyzing the Ni 2p spectrum of the passive layer reveals peaks corresponding to both metallic nickel and nickel hydroxide (Ni(OH)_2_) [65]. Additionally, the analysis of Mo 3d spectra identifies eight distinct peaks corresponding to Mo 3d_3/2_ and Mo 3d_5/2_, indicating different energy states associated with metallic molybdenum and its oxidation states: Mo^4+^, Mo^5+^, and Mo^6+^ [66]. The presence of Mo^6+^ and Mo^4+^ oxidation states in the film contributes to enhanced stability and an increase in Cr_2_O_3_ content [67]. Furthermore, the O 1s spectrum displays deconvoluted peaks for O^2−^, OH^−^, and H_2_O. Notably, the intensity of the O^2−^ peak, indicative of metal oxides in the passive film, is relatively lower than that of the OH^−^ peak, which is associated with metal hydroxides. The increase in Ti contents leads to a higher O^2−^/OH^−^ ratio, indicating a rise in the oxide components within passive films. This finding suggests that the introduction of Ti promotes the formation of a denser and more uniform passive film on the LMD-processed GH3536 alloy [68].

## 4. Discussion

### 4.1. Refinement Mechanism of Ti Addition on Solidification Structure

The experimental results reveal that the original GH3536 alloy sample processed by LMD exhibits coarse columnar grains with an average size of 36.31 μm. In contrast, the Ti-modified samples display fine equiaxed grains, with the average grain size reduced to 22.86 μm. This significant grain refinement due to the addition of Ti is closely associated with the enhanced corrosion resistance. This refinement phenomenon is primarily attributed to the solidification process, since there is no apparent solid-state phase transformation occurring within the GH3536 alloy [15,69]. The solidification of the Ti-modified GH3536 alloy takes place rapidly, leading to a non-equilibrium state during the nucleation and growth of grains [70]. During the solidification, a part of Ti atoms reacts with C and O to form Ti carbides and/or oxides, while the remaining Ti accumulates at the solid–liquid interface, as it does not fully convert into Ti compounds in a short solidification time. The following section will discuss the refinement mechanisms of the solidification structure by Ti alloying, concentrating on two key aspects: the mechanisms of heterogeneous nucleation and solute segregation.

In this study, the printing chamber was purged with argon gas to maintain a low oxygen level, specifically below 100 ppm. However, it is important to recognize that argon gas itself contains approximately 20 ppm of oxygen. This inherent oxygen content makes it challenging to completely eliminate air from the printing chamber using argon gas alone. In conventional steelmaking processes, aluminum is employed as a deoxidizer, allowing aluminum oxide (Al_2_O_3_) to rise to the surface of the molten metal and form the slag. In contrast, during the LMD process, the rapid cooling rate restricts the time available for Al_2_O_3_ particles to float to the melt surface. As a result, Al_2_O_3_ particles tend to be dispersed within the matrix of the GH3536 alloy. Notably, the observed grain refinement cannot be attributed to the presence of Al_2_O_3_ particles since the GH3536 alloy without Ti addition still exhibits a relatively large grain size. However, the Ti-modified GH3536 alloy samples show several Ti-modified Al_2_O_3_ particles. Previous studies [71] suggest that Ti compounds preferentially nucleate on the surfaces of existing oxides, such as Al_2_O_3_ particles, due to the significantly lower level of supercooling required for this process compared to homogeneous nucleation. These un-melted Al_2_O_3_ particles act as heterogeneous nucleation sites for Ti compounds, leading to the formation of Ti-modified Al_2_O_3_ particles dispersed throughout the deposition pool. During solidification, these Ti-modified Al_2_O_3_ particles serve as an effective nucleating agent for heterogeneous nucleation, facilitating grain refinement. Moreover, as the Ti content increases, the number of these Ti-modified Al_2_O_3_ particles increases, creating additional nucleation sites and further reducing the grain size. Alongside the role as effective nucleants, the restriction of growth driven by solute segregation is crucial for achieving the grain refinement. During the grain refinement process, the segregation of Ti atoms at grain boundaries can hinder grain growth and create a region of constitutional supercooling, which generates a driving force for enhanced nucleation [72]. This zone of constitutional supercooling limits grain growth while simultaneously promoting the activation of potent nucleants at grain boundaries. The substantial restrictions on growth enforced by the Ti solute slow down the movement of grain boundaries, thereby facilitating a greater number of nucleation events and ultimately resulting in refined grain structures.

### 4.2. Corrosion Resistance Mechanism of LMD-Processed GH3536 Alloy by Ti Alloying

In addition to the grain refinement achieved through Ti alloying, the Ti-modified GH3536 alloy exhibits a notable presence of LAGBs and low CSL grain boundaries. This formation enhances the distribution of grain boundary characteristics, thereby reducing the possibility of intergranular failure [32]. Specifically, CSL grain boundaries possess a higher density of junction points, which can disrupt the connectivity of HAGBs [40,73]. At these boundaries, a significant number of atoms occupy defined geometric positions, reflecting a low-energy state that contributes to the stability of the alloy [74]. The decrease in grain boundary energy is critical for enhancing the corrosion resistance of the material. Additionally, the refinement of grains is closely related to a reduction in passive current density and the formation of a more compact passive film. It is essential to note that grain boundaries possess higher energy levels and exhibit greater chemical reactivity compared to the bulk material. The increased grain boundary density enhances surface reactivity by facilitating electron activity and diffusion processes. This elevated reactivity, resulting from the higher density of grain boundaries, promotes the formation of additional nucleation sites for oxide films on the alloy surface, accelerating the development of a protective passive film. Moreover, the passive film formed on the GH3536 alloy samples exhibits semiconductor-like properties, with its stability being closely associated with the type of semiconductor and the density of charge carriers [75]. The Mott–Schottky analysis demonstrates that the passive films generated on GH3536 alloy samples processed by LMD have a bilayer structure, such as the Fe/Ni-rich outer layer as an n-type semiconductor and the Cr-rich inner layer as a p-type semiconductor. Both the donor density and acceptor density display a similar trend, decreasing to lower values of 3.31 × 10^21^ (1.64 × 10^21^) and 3.12 × 10^21^ (1.49 × 10^21^), respectively, at Ti contents of 0.2 wt.% and 0.3 wt.%. Due to the lower NA values indicated by the slopes of linear sections in Section II of Figure 12b, this study primarily focuses on the variation in ND values of the passive films. The decrease in ND values with the addition of Ti indicates that Ti contributes to reducing the presence of oxygen vacancies within the passive films. A reduction in oxygen vacancies after Ti addition is conducive to the creation of a denser and more cohesive passive film with enhanced protective properties [76], which significantly improves the corrosion resistance of LMD-processed GH3536 alloy samples.

## 5. Conclusions

This study investigates the corrosion resistance and stability of passive films of GH3536 alloy processed by LMD with varying Ti contents in a simulated PEMFC solution. The results indicate that the corrosion resistance of the LMD-processed GH3536 alloy significantly improves with increasing Ti content. However, when the Ti content exceeds 0.2 wt.%, the beneficial effect on corrosion resistance is weakened. Two primary mechanisms explain the enhanced corrosion resistance, involving the heterogeneous nucleation of Ti-modified Al_2_O_3_ and Ti solute segregation, which promotes the grain refinement. On the one hand, the optimized grain boundary characteristic (a high density of LAGBs and CSL grain boundaries) can effectively inhibit corrosion behavior. On the other hand, the grain refinement induced by Ti alloying can provide more active sites for Cr to initiate the formation of Cr_2_O_3_ and reduce the diffusion channel of Cr atoms to the passive film surface, thereby accelerating the enrichment of Cr within the passive film and enhancing the corrosion resistance of the GH3536 alloy.

## Figures and Tables

**Table 1:** Chemical composition of GH3536 alloy powder in this study (wt.%).

C	Si	Mn	Cr	W	B	Co	Mo	Fe	Ni
0.056	0.02	0.02	21.87	1.05	0.003	1.23	9.12	18.20	Bal.

**Table 2:** Parameters of electrochemical impedance spectroscopy components of LMD-processed GH3536 alloy samples with various contents of Ti.

Samples	*R*_S_ (Ω cm^2^)	*R*_f_ (Ω cm^2^)	*Y*_f_ (Ω^−1^·s^n^·cm^2^)	*n*_f_	*R*_ct_ (Ω cm^2^)	*Y*_dl_ (Ω^−1^·s^n^·cm^2^)	*n*_dl_	*X* ^2^	Errors
0.0 wt.% Ti	2.022				5.38 × 10^4^	1.09 × 10^−4^	0.8646	1.641 × 10^−4^	1.81%
0.1 wt.% Ti	1.775				6.57 × 10^4^	1.434 × 10^−4^	0.8341	1.162 × 10^−4^	2.31%
0.2 wt.% Ti	2.648	9870	2.318 × 10^−5^	0.647	9.89 × 10^4^	0.679 × 10^−4^	0.9161	4.378 × 10^−4^	2.09%
0.3 wt.% Ti	2.168	10,410	2.329 × 10^−5^	0.5602	0.12 × 10^4^	0.600 × 10^−4^	0.8857	3.571 × 10^−4^	1.89%

**Table 3:** The slope values derived from double-logarithmic plots of current–time for LMD-processed GH3536 alloy samples with various contents of Ti.

Sample	0.0 wt.% Ti	0.1 wt.% Ti	0.2 wt.% Ti	0.3 wt.% Ti
Initial stage	−0.61	−0.63	−0.79	−0.78
After 100 s	−0.37	−0.41	−0.60	−0.59

**Table 4:** Binding energies of the primary compounds of C, O, Ni, Cr, Fe and Mo.

Orbital	Peak (0.0 wt.% Ti)	Binding Energy	Peak (0.2 wt.% Ti)	Binding Energy
C 1s	C-C	1s: 284.80	C-C	1s: 284.80
	C-O	1s: 285.68	C-O	1s: 286.00
	C=O	1s: 288.60	C=O	1s: 288.69
O 1s	O^2−^	1s: 529.94	O^2−^	1s: 529.87
	OH^−^	1s: 531.63	OH^−^	1s: 531.52
	H_2_O	1s: 533.37	H_2_O	1s: 532.50
Ni 2p	Ni	2p_3/2_: 852.39	Ni	2p_3/2_: 852.38
		2p_1/2_: 869.39		2p_1/2_: 869.49
	Ni(OH)_2_	2p_3/2_: 855.78	Ni(OH)_2_	2p_3/2_: 855.77
		2p_1/2_: 873.98		2p_1/2_: 873.63
Cr 2p	Cr	2p_3/2_: 573.54	Cr	2p_3/2_: 573.59
		2p_1/2_: 582.77		2p_1/2_: 582.92
	Cr_2_O_3_	2p_3/2_: 575.79	Cr_2_O_3_	2p_3/2_: 575.82
		2p_1/2_: 585.29		2p_1/2_: 585.42
	Cr(OH)_3_	2p_3/2_: 577.10	Cr(OH)_3_	2p_3/2_: 576.97
		2p_1/2_: 586.86		2p_1/2_: 586.75
Fe 2p	Fe	2p_3/2_: 706.56	Fe	2p_3/2_: 706.62
		2p_1/2_: 719.51		2p_1/2_: 719.58
	FeO	2p_3/2_: 709.07	FeO	2p_3/2_: 708.40
		2p_1/2_: 721.87		2p_1/2_: 721.22
	Fe_2_O_3_	2p_3/2_: 711.51	Fe_2_O_3_	2p_3/2_: 711.09
		2p_1/2_: 725.31		2p_1/2_: 724.70
Mo 3d	Mo	3d_5/2_: 227.25	Mo	3d_5/2_: 227.34
		3d_3/2_: 230.32		3d_3/2_: 230.30
	Mo^4+^	3d_5/2_: 228.60	Mo^4+^	3d_5/2_: 228.48
		3d_3/2_: 231.95		3d_3/2_: 231.90
	Mo^5+^	3d_5/2_: 230.89	Mo^5+^	3d_5/2_: 230.89
		3d_3/2_: 233.94		3d_3/2_: 233.73
	Mo^6+^	3d_5/2_: 232.19	Mo^6+^	3d_5/2_: 232.18
		3d_3/2_: 235.24		3d_3/2_: 235.22

## Data Availability

The original contributions presented in this study are included in the article. Further inquiries can be directed to the corresponding author.
